# 2-Arachidonoylglycerol Synthesis: Facile and Handy Enzymatic Method That Allows to Avoid Isomerization

**DOI:** 10.3390/molecules27165190

**Published:** 2022-08-15

**Authors:** Roberta Ottria, Silvana Casati, Paola Rota, Pierangela Ciuffreda

**Affiliations:** 1 Dipartimento di Scienze Biomediche e Cliniche, Università degli Studi di Milano, Via G.B. Grassi 74, 20157 Milano, Italy; 2 Dipartimento di Scienze Biomediche, Chirurgiche e Odontoiatriche, Università degli Studi di Milano, Via della Commenda 10, 20122 Milano, Italy

**Keywords:** 2-Arachidonoylglycerol, biocatalysis, monoacylglycerol, *Mucor miehei* lipase

## Abstract

A simple and practical synthesis of 2-arachidonoyl glycerol (2-AG), an endogenous agonist for cannabinoid receptors, based on a two-step enzymatic process and a chemical coupling, was achieved with a good yield and negligible amount of the isomerization product 1-AG. Commercial preparation of immobilized lipase from *Mucor miehei* (MML) was selected as the most suitable enzyme to catalyze the efficient protection of glycerol using vinyl benzoate as an acyl transfer reagent in tetrahydrofuran. The same enzyme was used to remove the protective groups in positions 1 and 3. Owing to the mild neutral conditions and easy suitability of the method, 2-AG was obtained without any isomerization to the more stable 1-AG and air oxidation of acid chain. The synthetic method proposed here allows us to easily obtain 2-AG from the protected precursor in a one-step reaction without purification requirement.

## 1. Introduction

**Figure 1:**
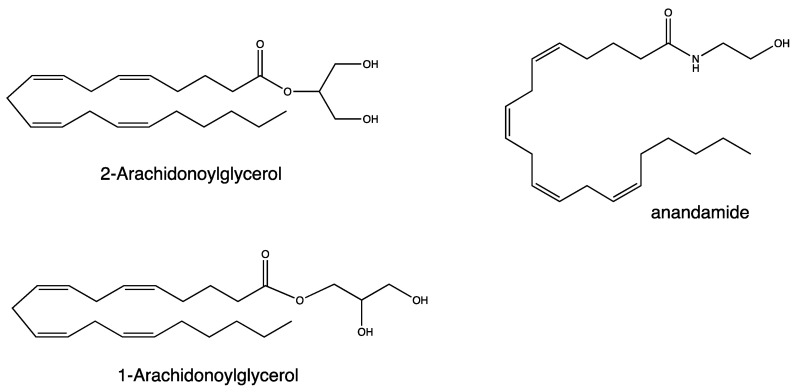
Structure of 2-Arachidonoylglycerol (2-AG), 1-Arachidonoylglycerol (1-AG), *N*-arachidonoylethanolamine (anandamide).

2-Arachidonoylglycerol (2-AG) is a monoacylglycerol (MAG) molecule containing an esterified arachidonic acid chain at the sn-2 position of the glycerol backbone (Figure 1). Together with structurally similar *N*-arachidonoylethanolamine (anandamide), 2-AG has been extensively studied as an endogenous ligand of cannabinoid receptors (CB1 and CB2) in the brain and other mammalian tissues and plays several physiological functions in both central and peripheral organs [1]. During either acute or chronic pathological conditions, the tissue and plasma levels of 2-AG, as well as of the other endocannabinoids (EC) or *N*-acylethanolamides (NAEs), are altered. The modulation of circulating or tissue EC and NAEs levels produces changes in cannabinoid receptor activation that acts to re-establish the homeostasis of other mediators, such as γ-aminobutyric acid and glutamate, and to restore the cell to its original steady-state condition [2]. Due to the prominent role of these signaling molecules, the literature reports numerous biochemical studies on the EC and NAEs levels associated with pathological conditions [3,4,5,6,7] or on the modification of the endocannabinoid system activity in cellular [8,9,10] or animal [11,12] pathological models. Moreover, the literature reports different LC or GC-MS methods that quantify EC and NAEs to study and understand their role in pathological processes and homeostasis restoration [3,7,13]. The importance of having pure and stable analytical standards for EC and NAEs is clear.

Chemical synthesis of 2-AG would enable ready access to this ligand for extensive study to elucidate the functions of this major neurochemical signaling molecule in biological and biochemical studies. However, the synthesis, isolation, storage, and testing of 2-AG are all compromised by the instability of the compound to both air oxidation, affording olefin rearranged hydro-peroxides, and ester rearrangement, affording 1-arachidonoyl glycerol (1-AG). Indeed, the four double bonds present in the arachidonoyl chain render 2-AG susceptible to oxidation processes prompted by drastic reaction conditions. Moreover, the main issue in the synthesis of pure 2-AG is the rapid migration of the arachidonoyl group from the secondary to the primary hydroxyl group of the glycerol, resulting in the formation of more stable 1-AG. The 1-MAG isomeric conformation is so thermodynamically stable that, at equilibrium, the acyl migration reaches a 1:9 2-MAG:1-MAG ratio [14,15]. This rearrangement to 1-AG, or 1-acyl glycerol derivatives in general, occurs under different reaction conditions, including temperature, solvent polarity, water activity, fatty acid chain length, acid/base impurities, time, and even in storage conditions at room temperature in various biologically compatible solvents. These variables must be considered during purification, analysis, and subsequent reactions using 2-MAG as an intermediate. Furthermore, the separation of these isomers is ineffective on a preparative scale, leaving mixtures that could compromise the interpretation of biological study results. Commercially available analytical standards indeed are often sold as organic solvent solutions, principally acetonitrile solutions, with a purity of up to 98% and 9:1 ratio 2-AG:1-AG. Very low amounts of pure 2-AG are also commercially available, but even in this case the presence of 1-AG is around 10% and the stability of the standard is guarantee only for 2–3 months.

To date, 2-AG is synthesized by either chemical [16,17,18,19] or chemoenzymatic methods [20,21,22,23]. The chemical methods described for the synthesis of 2-AG are based on the same procedure: acylation of suitable 1,3-protected glycerol, or glycidol, precursors with an activated arachidonic acid derivative, followed by deprotection and separation of the isomeric arachidonoylglycerols. Han and co-workers [24] use the triisopropylsilyl protecting group (TIPS) for the 1- and 3-hydroxyl functions of glycerol that can be removed by prolonged treatment with a solution of tetra-n-butylammoniofluoride (TBAF), buffered with acetic acid, at low temperatures. Prolonged treatment required for protecting group cleavage leads to 2 to 1 partial AG isomerization and increases the chance of arachidonic chain auto-oxidation. In the two other approaches 1,3-benzylidene-glycerol is used as substrate and, after the introduction of the arachidonoyl part, the acid-labile benzylidene group of the 1,3-benzylidene-2-acylglycerols is separated by reaction with boric acid; the resulting mixture of the 1,3- and 1,2-phenylboronate esters are hydrolyzed by washing with water to produce 2-monoglycerides [18,25]. However, the protecting groups’ cleavage with boric acid proved difficult to complete and undergoes increasing isomerization to 1-AG after the longer reaction time required. The use of phenylboronic acid instead of boric acid for the cleavage of benzylidene acetal gave better results, leading to a cleavage and to the formation of 13% of 1-arachidonoylglycerol 2,3-phenylboronate ester [26], that being the least subject to rearrangement in the final deblocking phase (<1% as determined by HPLC or ^1^H NMR analysis) leading to a total yield of 65%. Another synthesis of 2-AG used, as starting material, glycidyl arachidonate obtained from glycidol and commercially available arachidonic acid [16]. The transformation of glycidyl arachidonate into the 2-AG derivative is promoted by trifluoroacetic anhydride. 1,3-Dibenzyloxy-2-propanol was chosen as 1,3 protected glycerol, because it is possible to remove the protecting groups in a clean and quantitative manner using B-chlorocatecholborane as a selective reagent [17].

B-Chlorocatecholborane was also used for acetal cleavage of cis-arachidonoylbenzylidene glycerol [19] that demonstrated the mildness of the reagent on an especially sensitive transformation while also affording an improved quality of product of current interest. Nevertheless, the silyl [24] and benzyl [17,19] protected glycerol approaches experienced at least 5% rearrangement, demonstrating the sensitivity of this molecule to deblocking conditions and identifying it as a probe of the mildness of reaction conditions.

The enzymatic methods described for the synthesis of 2-AG are based on a two-step enzymatic process [20,21,22,23]. 1,3-Dibutyrylglycerol is synthesized by selective esterification of glycerol, using vinyl butyrate in the presence of immobilized *Candida antarctica* lipase B and anhydrous dichloromethane as solvent. The resulting butyryl groups act to protect these sites for the subsequent arachidonate ester placement at the free sn-2 position to form 2-arachidonoyl-1,3 dibutyrylglycerol. Next, the butyryl groups are selectively removed in a lipase-mediated transesterification reaction to yield 2-AG. Even if the use of immobilized *Candida antarctica* lipase B allows milder reaction conditions, these methods are time consuming, lead to byproduct formation, require the following purification step after every reaction step, and show isomerization of around 10%. Using immobilized enzymes as biocatalysts is a well-known technique that allows enzyme reuse and often improves enzyme stability, biocompatibility, and resistance to environmental changes [27,28]. Moreover, they are more easily removed from the reaction mixture leading to handy synthetic procedures. However, the various enzyme immobilization methods, such as physical adsorption, entrapment or encapsulation, chemical cross-linking, and covalent binding, can differently affect enzyme stability, chemical or conformational structures or enzyme active sites surrounding solvent, and enzyme selectivity and specificity. [29]. Furthermore, interfacial activation of the lipase, the fixation of the lipase in its open (and active) form, is also modulated by the different immobilization methods and supports. Thus, having a “universal” optimal biocatalyst is far from straightforward. Nevertheless, having different immobilized biocatalysts with the same enzyme allows forproducing a large library of biocatalysts from a specific lipase [30].

Searching for an alternative and efficient methodology that would bypass these problems during 2-AG synthesis, we have developed an enzyme-catalyzed efficient and highly regioselective synthesis that enables an easy release of 2-AG without rearrangement to 1-AG from a precursor that exhibits resistance to oxidation and ester rearrangement during storage.

## 2. Results and Discussion

**Scheme 1:**
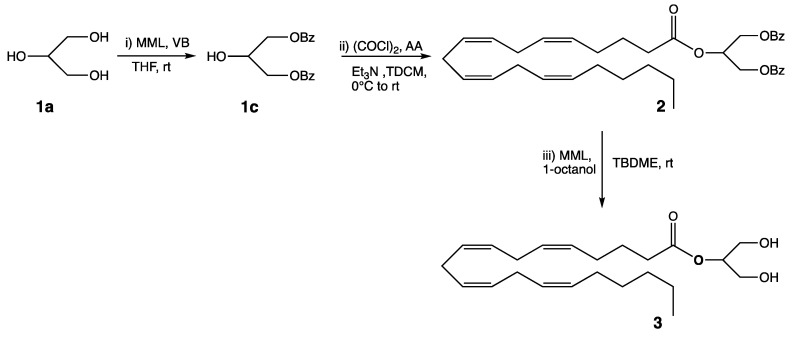
Efficient synthesis of 2-AG; (i) **1a**/MML 1/3, vinyl benzoate (VB) 2.5 eq, room temperature (rt), 2.5 h, >98% yield; (ii) oxalylcloride (2 eq), arachidonic acid (AA, 1 eq), Et_3_N, rt, 5 h, 85%; (iii) **2**/MML 1/3, 1-octanol (9 eq), rt, 24 h, >98% yield, isomerization <1%.

With the aim of obtaining a new synthetic route for 2-AG able to completely overcome isomerization to 1-AG, we explored the use of lipase enzymes for the protection and the deprotection steps. The optimal conditions were selected to avoid time-consuming purification steps and 2-AG to 1-AG isomerization. We planned a three-step procedure according to Scheme 1. We optimized the enzymatic synthesis of 1,3-dibenzoyl glycerol **1c** starting from glycerol. Then, compound **1c** was coupled with arachidonic acid (AA) to give the protected 2-AG **2**. Finally, we set up an enzymatic alcoholysis reaction to achieve 2AG, compound **3**, with yields higher than 98% and highly regioselective.

**Figure 2:**
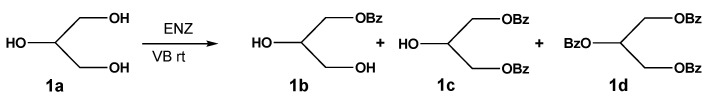
General reaction and products of enzymatic benzoylation used as standards for GC analysis. (Enzyme (ENZ), Room Temperature (rt)).

We started our protocol with the 1,3-dibenzoyl glycerol **1c** synthesis optimization, starting from glycerol. The effects of solvent, acyl transfer agent, lipase used, amount of enzyme, reagents ratio, and reaction time on the 1,3-di-benzoylation yield were examined during the protection glycerol step. A gas chromatographic (GC) analysis method was set up to follow the reaction and to quantify and qualify the reaction products. Better separation between glycerol **1a**, glycerol mono-benzoate (**1b**), glycerol 1,3-di-benzoare (**1c**), and glycerol tri-benzoate (**1d**) (Figure 2), protected 2-AG (**2**) and 2-AG (**3**) was obtained with a HP-50+ Agilent Technology column (12 m; 0.2 mm; 0.31 mm) in an analysis time of 14.5 min. The isomerization rate of 2-AG to 1-AG was then evaluated on purified products by NMR analysis [31]. As acyl transfer agent vinyl benzoate (VB) was selected. In general, a benzoic ester should be preferred as an acyl protecting group of polyhydroxy compounds, owing to the stability of benzoyl moiety, in general higher than that of other acyl derivatives, and to a less-pronounced tendency to vicinal migration [32]. Despite this, relatively few examples of selective enzymatic benzoylation of polyhydroxylated compounds are currently available. Moreover, our past experience in selective protection of polyhydroxy compounds [33] prompted us to select benzoate as the first protecting group to assess, which remained the only protecting group tested due to the optimal results obtained. 

The solvent affects the solubility of the reactant, and the product also influences the enzyme activity due to the differences in solvent polarity, with highly polar solvents resulting in the loss of lipase activity [34,35]. Other methods [20,21,22,23] report the use of dichloromethane (DCM) as a reaction solvent despite the very poor solubility of glycerol in this solvent. This issue slows down the reaction rate, leading to longer reaction times, reduced yields, and chromatographic purification steps requirements. Starting from these considerations, we assessed glycerol solubility in DCM, acetone, dioxane (DIOX), tetrahydrofuran (THF) and di-*tert*butyl-dimethyl ether (TBDME) with dioxane and tetrahydrofuran that displayed better results and the final exclusion of acetone and di-*tert*butyl-dimethyl ether from the following experiments. Finally, THF was selected for the screening of the lipase enzyme. Supported microbial lipases from *Pseudomonas cepacea* (PCL), *Mucor miehei* (MML), *Candida antarctica* (CAL), *Candida cylindracea* (or *Candida rugosa*, CCL), porcine pancreas lipase from (PPL), *Pseudomonas fluorescence* (PFL) and *Pseudomonas* sp. (PSL) were assessed at 1/1 enzyme/substrate ratio in THF using 2.5 equivalents of VB at room temperature. This first screening allowed us to exclude PPL, PFL, and PSL from future experiments due to their inability to create benzoylation products. MML, CCL and CAL benzoylation activity was then assessed in different solvents, namely DIOX, THF, and DIOX-DCM 1/1 mixture, to find better conditions considering glycerol solubility and enzyme activity. The reaction was monitored by GC and areas of the obtained selections were used to express the reaction mixture composition in percentage. Table 1 reports obtained percentages of glycerol, mono-, di- and tri-benzoyl glycerol after 72 h of reactions. Obtained results, performed with commercially available immobilized enzymes and in the described conditions, allowed us to select THF as solvent and MML, a well-known and extensively studied enzyme [36,37,38], as the enzyme with better performance. However, considering immobilization effects on enzyme activity as described in the introduction, it must be emphasized that because this study does not really compare PCL, MML, CAL, PPL and PFL enzymes, nor their different immobilization protocols and supports, it is possible that some of the discarded enzymes may greatly improve their properties when using other immobilization protocols. Unfortunately, the unavailable data on immobilization techniques used to produce commercial enzymes does not allow speculations on the mechanistic aspect of the reaction, making it very difficult to establish an accurate model that could explain the interesting results obtained.

**Figure 3:**
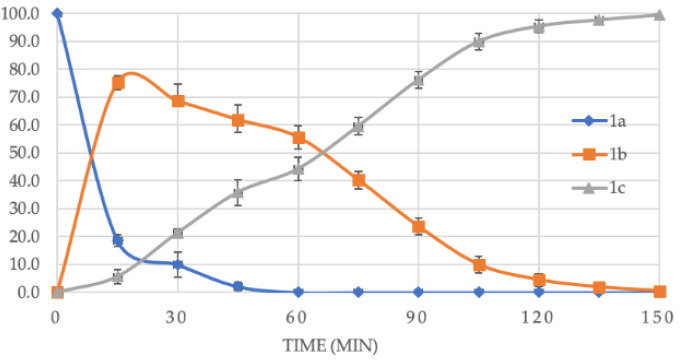
Time course of glycerol benzoylation performed with MML/**1a** (3/1), VB/**1a** (5/1) in THF at room temperature. Reaction progression was followed by GC and mixture composition is expressed in percentage. **1a** = Glycerol; **1b** = monobenzoyl glycerol; **1c** = 1,3-dibenzoyl glycerol.

After the enzyme selection, enzyme/substrate ratios and substrate/VB ratios were also investigated. Different experiments were performed, varying enzyme/substrate ratios from 1/1 to 1/5 and substrate/VB ratios from 1/1 to 1/10. Glycerol/MML 1/3 and glycerol/VB 1/5 were then selected, giving **1c** in 2.5 h and yield >98%. A time-course analysis of the glycerol benzoylation reaction in the optimized condition was performed, followed by GC, and is reported in Figure 3.

The 1,3-dibenzoyl glycerol **1c** was then coupled with arachidonic acid (AA) to give the protected 2-AG **2**. Several classical coupling reactions using different acid activators such as 1-(3-dimethyl-aminopropyl)-3-ethylcarbodiimide hydrochloride (EDC) or carbonyldiimidazole (CDI) or N,N′-dicyclohexylcarbodiimide (DCC) in presence of 4-dimethylaminopyridine (DMAP), 1-[bis(dimethylamino)methylene]-1H-1,2,3-triazolo [4,5-b]pyridinium 3-oxid hexafluorophosphate (HATU) or 1-[(1-(cyano-2-ethoxy-2-oxoethylideneaminooxy) dimethylaminomorpholino)] uronium hexafluorophosphate (COMU) in the presence of diisopropylethylamine (DIPEA), or the activation of the acidic moiety passing through chlorine using oxalylchloride, were assessed. At first, COMU, a molecule of a third-generation uronium-type coupling previously used for the synthesis of other EC and NAEs or their derivatives [39,40,41,42,43], was assessed for arachidonate ester placement in the sn-2 position. Unfortunately, this procedure gave low yields (<40%) associated with long reaction times (>24 h). The use of oxalyl chloride gave the best coupling performance, leading to compound **2** in high 85% yield, after flash chromatography. The lowering of the reaction yield using other acidic moiety activators was probably due to their steric hindrance in addition to that of the benzylidene protecting group.

**Figure 4:**
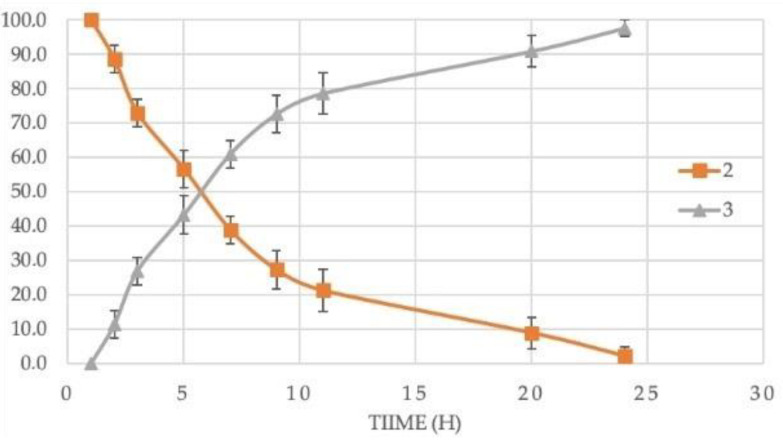
Time course of 2-AG alcoholysis performed with MML/**2** (3/1), 1-octanol/**2** (9/1) in TBDME at room temperature. Reaction progression was followed by GC and mixture composition is expressed in percentage. **2** = 1,3-dibenzoate-2-AG; **3** = 2-AG.

Finally, MML was also used to perform an alcoholysis reaction, and methanol, ethanol, and TBDME in the presence of 1-octanol were assessed as solvents. The use of MML/compound **2** ratio 3/1 in TBDME with nine equivalents of 1-octanol allowed us to obtain 2-AG (**3**) in a yield higher than 97% in 24 h. Then, the solvent, an immobilized MML, residual 1-octanol, and volatile by-products could be easily filtered off and/or evaporated under vacuum. NMR analysis of the crude allowed us to exclude the 1-AG formation and verify the purity of the obtained product. Figure 4 reports a time-course analysis of the alcoholysis reaction.

In summary, the improved and practical synthesis of 2-AG was successfully carried out without any isomerization to the formation of the more stable 1-arachidonoylglycerol. Indeed, the mild neutral conditions, easy scalability, and the possibility to bypass the last purification step, usually performed on silica in slightly acid conditions that favor isomerization, added advantages to the current method for synthesizing of 2-AG.

## 3. Material and Methods

### 3.1. General Information

Immobilized microbial lipases PCL, MML, CAL, CCL, PPL, PFL, and PSL were purchased from Sigma Aldrich (St. Louis, MO, USA). All reagents and solvents were also purchased from Sigma Aldrich (St. Louis, MO, USA) and used without prior purification. For the obtained intermediate **2** and the final compound **3** the ^1^H and ^13^C NMR assignments refer to this general structure:


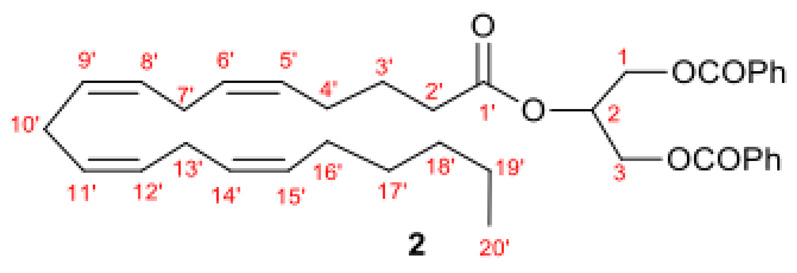


### 3.2. NMR Analysis

NMR spectra were registered on a Bruker AVANCE 500 spectrometer equipped with a 5 mm broadband reverse probe and deuterium lock with field z-gradient operating at 500.13 and 125.76 MHz for ^1^H and ^13^C, respectively. All NMR spectra were recorded at 298 K in CDCl_3_ (isotopic enrichment 99.98%) solution, and the chemical shifts were reported on a δ (ppm) scale. The central peak of DMSO-d_6_ signals (2.49 ppm for ^1^H and 39.50 ppm for ^13^C) and of CDCl_3_ signals (7.26 ppm for ^1^H and 77.2 ppm for ^13^C) were used as internal reference standard. Acquisition parameters for 1D were as follows: ^1^H spectral width of 5000 Hz and 32 K data points providing a digital resolution of ca. 0.305 Hz per point, relaxation delay 2 s; ^13^C spectral width of 29,412 Hz and 64 K data points providing a digital resolution of ca. 0.898 Hz per point, relaxation delay 2.5 s. The experimental error in the measured ^1^H-^1^H coupling constants was ±0.5 Hz.

### 3.3. Synthesis and Purification of Glycerol Mono-, Di-, and Tri-Benzoate as Standard for Gas Chromatography Analyses

The immobilized MML (500 mg) was added to a solution of glycerol (1.0 g, 10.8 mmol) and VB (8.04 g, 54.3 mmol) in dichloromethane (3 mL) at room temperature. The resulting mixture was stirred for 24 h. Then, additional lipase (400 mg) was added to the reaction mixture, which was stirred for an additional 12 h. The immobilized lipase was filtered off, the solvent was evaporated under reduced pressure, and the residue was chromatographed on silica (DCM/methanol 95:5) to yield mono, di, and tri 2-hydroxypropane-1,3-diyl benzoate as colorless oils. The TLC retention fractions (*Rf)* (DCM/methanol 95:5) are: 0.86 for 2-hydroxypropane-1,3-diyl tribenzoate, 0.67 for 2-hydroxypropane-1,3-diyl dibenzoate and 0.56 for 2-hydroxypropane-1,3-diyl monobenzoate. NMR spectroscopy, as applied before [36,37,38], was used to investigate the structure and purity of obtained compounds. NMR analyses, performed in CDCl_3_, confirmed the structure and purity of the three molecules.

### 3.4. Gas Chromatography Separation of Glycerol Mono-, Di-, and Tri-Benzoate

Aliquots from the reaction mixture were withdrawn at different time points, diluted in diethyl ether, and injected in a gas chromatograph using a Hewlett Packard GC System HP6890 coupled with FID detector and using an HP-50+ Agilent Technology column (12 m; 0.2 mm; 0.31 mm). The separation was obtained with the following method: 80 °C to 240 °C 20 °C/min, 240 °C for 0.5 min; 240 °C to 260 °C 10 °C/min, 260 °C for 1 min; 260 °C to 280 °C 5 °C/min, 280 °C for 0.5 min in a total time analysis of 14.5 min. The Retention times (*Rt*) obtained are listed below: Glycerol (**1a**) *Rt* = 3.2 min; monobenzoate (**1b**) *Rt* 4.5 min; dibenzoate (**1c**) *Rt* = 7.4 min, tribenzoate (**1d**) *Rt* = 10.3 min; 2-AG (**3**) *Rt* = 12.9 min; 2-AG-1,3- dibenzoate (**2**) *Rt* = 14.2 min. Obtained selection areas were used to express the reaction mixture composition in percentage.

### 3.5. Synthesis of 2-Hydroxypropane-1,3-diyl dibenzoate (**1c**)

Immobilized MML (1.50 g) was added to a solution of glycerol (1.0 g, 18.8 mmol) and vinyl benzoate (6.2 g, 31.0 mmol) in THF (10 mL) at room temperature. The resulting mixture was stirred for 2.5 h. The lipase was filtered off and the solvent was evaporated off under reduced pressure to give 96% yield as a colorless oil. ^1^H NMR (500 MHz, CDCl_3_) δ 8.05 (4H, d, *J* = 7.8 Hz, H*_ortho_*), δ 7.57 (2H, t, *J* = 7.8 Hz, H*_para_*), δ 7.44 (4H, d, *J* = 7.8 Hz, H*_meta_*), 4.58–4.51 (4H, m, 1 and 3-CH_2_), 4.43–4.38 (1H, m, 2-CH_2_); ^13^C NMR (CDCl_3_) δ 166.8 (CO), 133.5 (C*_para_*), 130.3 (C*_aromatic_*), 129.9 (C*_ortho_*), 128.8 (C*_meta_*), 68.7 (2-CH), 66.0 (1- and 3-CH_2_).

### 3.6. Synthesis of 2-((5Z,8Z,11Z,14Z)-Icosa-5,8,11,14-tetraenoyloxy)propane-1,3-diyl dibenzoate (**2**)

To a solution of arachidonic acid (110 mg, 0.37 mmol) and Et_3_N (1 eq) in anhydrous DCM (3 mL), cooled by an ice bath, oxalylchloride (2 eq) was slowly added and the reaction mixture stirred for 5h. The obtained chloride was then reacted with 1,3-dibenzoylglycerol (**1**) (75 mg, 0.37 mmol), without further purification, in anhydrous DCM containing Et_3_N (0.07 mL, 0.5 mmol) and the solution was stirred for 24 h. The solvent was then evaporated and crude was purified by flash chromatography (hexane/acetone; 7:3) to afford pure triglyceride as colorless oil (1.06 g, 85%). *Rf* 0,64 (hexane/acetone; 7:3). IR (neat) 3012, 2931, 1741, 1456, 1167, 1094 cm^−1^; ^1^H NMR (CDCl_3_) 8.06 (4H, d, *J* = 7.8 Hz, H*_ortho_*), δ 7.57 (2H, t, *J* = 7.8 Hz, H*_para_*), δ 7.45 (4H, d, *J* = 7.8 Hz, H*_meta_*), 5.54–5.48 (m, 1H, 2-CH), 5.32–5.50 (8H, m, 5′- 6′- 8′- 9′- 11′- 12′- 14′- 15′-CH), 4.75–4.45 (4H, m, 1 and 3-CH_2_), 2.82–2.88 (6H, m, 7′- 10′- and 13′-CH_2_), 2.64 (2H, t, *J* = 7.0, 2′-CH_2_), 2.24 (2H, dt, *J* = 7.0, 7.4, 4′-CH_2_), 2.08 (2H, dt, *J* = 7.6, 7.6, 16′-CH_2_), 1.87 (2H, tt, *J* = 7.0, 7.6, 3′-CH_2_), 1.28–1.39 (6 H, m, 17′-, 18′-, 19′-CH_2_), 0.90 (3H, t, *J* = 7.6, 20′-CH_3_); ^13^C NMR (CDCl_3_) δ 172.8 (1′-CO), 166.1 (CO), 133.3, 130.5, 129.7, 129.5, 129.5, 128.7, 128.5, 128.3, 128.0, 127.8, 127.5 (5′, 6′, 8′, 9′, 11′, 12′, 14′, 15′, C*_para_*, C*_ortho_*, C*_meta_*), 69.1 (2-CH), 62.3 (1 and 3-CH_2_), 33.7 (2′), 31.5 (18′), 29.3 (17′), 27.2 (16′), 26.5 (4′), 25.6 (7′, 10′, 13′), 24.7 (3′), 22.6 (19′), 14.1 (20′).

### 3.7. Synthesis of (5Z,8Z,11Z,14Z)-1,3-Dihydroxypropan-2-yl Icosa-5,8,11,14-tetraenoate (**3**)

Triglyceride **2** (50 mg, 0.085 mmol) was dissolved in TBDME containing 1-octanol (100 mg, 0.918 mmol) at room temperature; the reaction was started by the addition of immobilized MML (150 mg), and the reaction mixture was maintained under mechanical agitation for 24h until the starting material was completely consumed. After completion of the reaction the immobilized enzyme was filtered off and washed with ether, the solvent was evaporated, and 2-AG **3** (31.5 mg, 98%) was obtained as a colorless oil. IR (neat) 3420, 3012, 2927, 1736, 1456, 1378, 1277, 1152 cm^−1^; ^1^H NMR (500 MHz, CDCl_3_) δ 5.42–5.33 (8H, m, 5′- 6′- 8′- 9′- 11′- 12′- 14′- 15′-CH), 4.21 (2H, dd, *J* = 11.7, 4.5 Hz, 1 or 3-CH_2_), 4.15 (2H, dd, *J* = 11.7, 6.2 Hz, 3 or 1-CH_2_), 3.95–3.91 (1H, m, 2-CH), 2.85–2.79 (6H, m, 7′- 10′- and 13′-CH_2_), 2.13 (2H, t, *J* = 7.0, 2′-CH_2_), 2.06 (2H, dt, *J* = 7.0, 7.4, 4′-CH_2_), 1.72 (2H, dt, *J* = 7.6, 7.6, 16′-CH_2_), 1.87 (2H, tt, *J* = 7.0, 7.6, 3′-CH_2_), 1.38–1.24 (6 H, m, 17′-, 18′-, 19′-CH_2_), 0.89 (3H, t, *J* = 7.6, 20′-CH_3_); ^13^C NMR (CDCl_3_) δ 172.8 (1′-CO), 130.7, 129.2, 128.9, 128.8, 128.5, 128.2, 128.0, 127.7 (5′, 6′, 8′, 9′, 11′, 12′, 14′, 15′), 70.4 (2-CH), 65.4 (1 and 3-CH_2_), 31.7 (2′), 31.5 (18′), 29.8 (17′), 27.4 (16′), 26.6 (4′), 25.6 (7′, 10′, 13′), 24.9 (3′), 22.7 (19′), 14.1 (20′). The ^1^H and ^13^C spectral data (500.13 and 125.76 MHz, CDCl_3_) are in agree with literature values [21].

## 4. Conclusions

In this work, we developed a synthetic strategy, based on only three steps, that overcomes the two main problems associated with the preparation of the 2-AG, (i) the rearrangement of this compound to the more stable 1-AG isomer, and (ii) the air oxidation of the arachidonic acid chain. Moreover, the present procedure allows for minimizing by-products formation, giving good yields at any step. In particular, the very mild conditions of the deprotection step, performed at room temperature with the commercial preparation of the immobilized MML that can be easily eliminated by filtration, allow obtaining 2-AG in a purity grade that does not requires further purification, overcoming the problem of isomerization (10–15%) that often occurs also during this step due the slightly acidic conditions of flash chromatography. Moreover, the procedure presented for alcoholysis here is simple and can be performed in any laboratory, without the need for organic synthesis expertise or special equipment. This issue is of fundamental importance, because it could render pure analytical standards accessible to analytical laboratories that could store the protected 2-AG or labeled 2-AG, and obtain the more unstable pure standard only when required for the analysis.

## Figures and Tables

**Table 1:** Effects of solvent on the benzoylation of glycerol performed by MML, CCL and CAL. Percentage of glycerol (**1a**), mono-(**1b**), di-(**1c**) and tri-benzoyl glycerol (**1d**) present in the reaction mixture after 72 h.

	DIOX				DIOX-DCM				THF			
	1a	1b	1c	1d	1a	1b	1c	1d	1a	1b	1c	1d
**MML**	7	80	13	-	8	90	2	-	10	51	39	-
**CCL**	16	82	2	-	21	76	2	-	18	58	25	-
**CAL**	100	-	-	-	54	38	8	-	36	52	18	-

## Data Availability

Not applicable.

## Sample Availability

Samples of the compounds are not available from the authors.
